# Analgesic Effects of Interferential Current Therapy: A Narrative Review

**DOI:** 10.3390/medicina58010141

**Published:** 2022-01-17

**Authors:** Érika Patrícia Rampazo, Richard Eloin Liebano

**Affiliations:** Physiotherapeutic Resources Research Laboratory, Department of Physical Therapy, Federal University of São Carlos (UFSCar), Sao Carlos 13565-905, SP, Brazil; erika.rampazo@gmail.com

**Keywords:** interferential current, kilohertz-frequency alternating current, medium-frequency alternating currents, burst-modulated alternating currents, electrical stimulation, physical therapy modalities, electroanalgesia

## Abstract

*Background and Objectives*: Transcutaneous electrical stimulation of low- and medium-frequency currents is commonly used in pain management. Interferential current (IFC) therapy, a medium frequency alternating current therapy that reportedly reduces skin impedance, can reach deeper tissues. IFC therapy can provide several different treatment possibilities by adjusting its parameters (carrier frequency, amplitudemodulated frequency, sweep frequency, sweep mode or swing pattern, type of application (bipolar or quadripolar), time of application and intensity). The objective of this review article is to discuss the literature findings on the analgesic efficacy of IFC therapy. *Conclusions*: According to the literature, IFC therapy shows significant analgesic effects in patients with neck pain, low back pain, knee osteoarthritis and post-operative knee pain. Most of the IFC parameters seem not to influence its analgesic effects. We encourage further studies to investigate the mechanism of action of IFC therapy.

## 1. Introduction

Interferential current (IFC) therapy is a simple, non-invasive and non-pharmacological treatment commonly used in clinical practice to alleviate pain, mainly of musculoskeletal origin, for muscle strength production, edema reduction, autonomic effects (control of incontinence, heart rate variability, blood flow velocity and vessel size), tissue repair and spasticity treatment after a cerebrovascular accident [1,2,3,4,5,6], mostly in the United Kingdom, other European countries and Australia [7]. It is a medium-frequency alternating current, and although commercial units allow several parameter adjustments, the rationale behind each parameter effect has been based on textbooks and the clinical experience of physical therapists rather than well-controlled studies [8].

Skin impedance (resistance) is inversely proportional to the frequency of an applied current [9]. The fundamental aspects of IFC therapy involve reducing cutaneous nerve stimulation and maximizing the current that permeates the tissues [3] with a higher carrier frequency, making it more suitable for treating deeper tissue layers [10]. Below, we present the definition, history, physiological effects and parameters of IFC therapy. The IFC units contain several parameters that can be adjusted, and our aim is to show the differences for each parameter.

## 2. Methods

In this narrative review, a literature search was performed using PubMed, Embase and PEDro to identify studies reporting on the definition, physiological effects, parameters and analgesic efficacy of IFC therapy. The following search terms were used: interferential current, medium-frequency currents, burst-modulated alternating current, medium-frequency alternating currents, electric stimulation therapy and electrotherapy. Additional studies were located through a review of the reference lists of the above articles and through personal searches.

## 3. Definition

**Figure 1:**
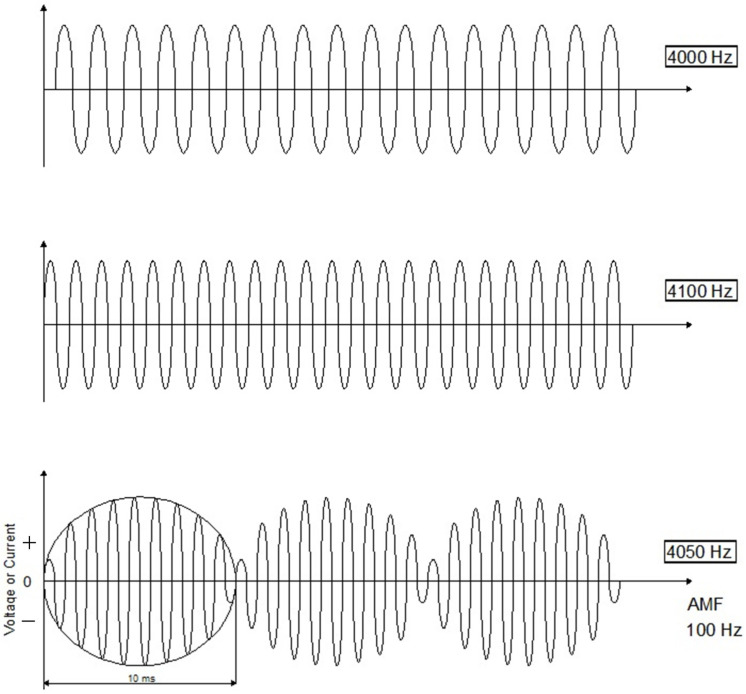
The two out-of-phase medium-frequency alternating currents (4000 Hz and 4100 Hz) “interfere” within the tissues and produce an amplitude-modulated frequency (AMF) of 100 Hz. The resulting frequency is 4050 Hz, and the duration of each burst is 10 milliseconds (ms). Hz: hertz.

IFC therapy involves the transcutaneous application of two out-of-phase medium-frequency alternating currents (>1 kHz to < 10 kHz) with a view to delivering currents to deep-seated tissue. For example, one of the alternating currents may be a fixed frequency at 4000 Hz, while the frequency of the other alternating current can be set between 4000 and 4250 Hz. The two medium-frequency currents “interfere” within the tissues and produce an amplitude-modulated low “beat” frequency (0–250 Hz), which is the difference between the values of the two currents applied [9] (Figure 1).

The IFC is an example of a burst-modulated alternating current (BMAC) with sinusoidal modulation, also known as kilohertz-frequency alternating current, and is reportedly more comfortable, reaches deeper tissues and induces greater muscle torque than low-frequency pulsed currents [7]. Nevertheless, it is important to exercise caution in relation to muscle force production, because Bellew et al. (2012) observed that the IFC actually produced a higher percentage of maximal voluntary isometric force of the knee extensors in healthy subjects when compared to the Russian current; however, there was no significant difference between the IFC and burst-modulated low-frequency biphasic pulsed currents [11].

## 4. History

IFC therapy was developed by Dr. Hans Nemec in the early 1950s in Vienna and has since been extensively used in Europe (Austria, Germany, Bulgaria and France). The aim of crossing two medium-frequency alternating currents was to utilize the concept that the skin offers little ohmic resistance to the passage of medium frequencies (two digits), whereas direct currents and low frequencies encounter very high ohmic resistance (between 2000 and 4000 ohms) [12,13].

## 5. Physiological Effects of IFC Therapy

Although IFC therapy has been used for the past several decades, the physiological effects are not established enough to explain the analgesic effect [14]. Goats (1990) reported that IFC therapy decreases the stimulation of cutaneous sensory nerves close to the electrodes while increasing the effect on deep tissues [3]. Some theories have been proposed to explain the analgesic effect, such as the gate control theory, descending pain suppression pathway, physiological blockage and placebo effect [14].

- Gate control theory of pain perception: Proposed by Melzack and Wall in 1965 [15], it has been suggested that an IFC frequency of 100 Hz can activate the large-diameter, low-threshold nerve fibers and that it uses the “pain-gating” system to achieve analgesia [14].
- Descending pain suppression pathway: Involves endogenous opioid (endorphins, dynorphins and enkephalins) release from periaqueductal grey matter (PAG) and the rostral ventral medulla (RVM) (nucleus raphe magnus (NRM), reticular nuclei and the spinal dorsal horn) [14,16]. These endogenous opioids play an important role in the control of nociceptive messages from primary afferent nerves [14]. A pulse duration range of 100–200 µs may activate large-diameter fibers, once their threshold is lower than that of the small-diameter A-delta and C fibers. Most IFC devices have a fixed pulse duration of 125 µs. However, it is not clear how IFC therapy can selectively activate the different nerve fiber types [14].
- Physiological blockage (Wedensky inhibition): C and Aδ fibers may not conduct nociceptive impulses after frequency stimuli greater than approximately 15 Hz and 40 Hz, respectively [3,14].
- Placebo effect: Refers to the patient–therapist relationship and types of modalities used during treatment. IFC devices are technically advanced and visually impressive, which may help convince patients that they are receiving an effective treatment [14].

It is important to highlight that to date, there has been no experimental research investigating the mechanism of action of IFC therapy for pain relief. IFC therapy has often been used for pain disorders; however, there is a lack of literature to explain its use. Some authors have based their claims on studies that investigated the mechanism of action of TENS. Goats et al. (1990) claimed that IFC therapy has a powerful placebo effect [3]. Thus, studies are needed to investigate the analgesic mechanism of action of IFC therapy. In addition, it is important to conduct more randomized clinical trials investigating the temporal summation of pain or conditioned pain modulation to assess the pain modulatory system of IFC therapy, such as in studies that have investigated other therapies [17,18].

## 6. IFC Parameters

There are several parameters that can be adjusted in IFC devices, including the carrier frequency, amplitude-modulated frequency (AMF), sweep frequency, sweep mode or swing pattern (slope), application type (bipolar or quadripolar application) and application time and intensity. There are a wide range of options for each one, all of which can be adjusted.

Most therapists select IFC parameters by trial and error, and some researchers believe that IFC therapy may be effective for a variety of diseases; the interference wave mediates the physiological and clinical effects, while different physiological effects are produced according to different amplitude-modulated wave frequencies [19]. Some of these claims about the effectiveness of IFC therapy have been based on personal experience [19]. Given the wide variety of parameters, it is important to understand the different effects of varying dosages, while the choice of parameters should be based on the findings of randomized clinical trials and systematic reviews. As such, it is relevant to discuss the current scientific evidence.

### 6.1. Carrier Frequency

The carrier frequency refers to a medium-frequency alternating current or kilohertz-frequency alternating current [1]. This frequency range of 1 to 10 kHz can be adjusted in modern IFC devices [20]. Some studies have investigated the effects of different carrier frequencies:

- Venancio et al. (2013) studied the effects of the carrier frequency on the pressure pain threshold (PPT) and sensory comfort in healthy subjects. One hundred and fifty subjects were randomly allocated to 1 of 5 groups according to carrier frequency (1 kHz, 2 kHz, 4 kHz, 8 kHz and 10 kHz). An AMF of 100 Hz was used for 20 min with two self-adhesive electrodes placed on the lateral aspect of the forearm, and the current amplitude was increased until a strong but comfortable paresthesia was reached. The authors found that a 1 kHz carrier frequency increased the PPT during and after stimulation compared to 8 kHz and 10 kHz. In addition, carrier frequencies of 1 kHz and 2 kHz were more uncomfortable compared to those of 4, 8 and 10 kHz [20];
- Correa et al. (2016) tested the analgesic effects of IFC (1 kHz and 4 kHz) in nonspecific chronic low back pain. One hundred and fifty subjects were randomly allocated to 1 of 3 groups (1 kHz, 4 kHz and placebo). The IFC parameters were: 1 or 4 kHz, AMF of 100 Hz, sweep frequency of 50 Hz, 1:1 sweep mode/swing pattern (slope), 30 min of stimulation with four electrodes (5 × 9 cm) on the lumbar region. Both carrier frequencies reduced analgesic consumption and increased PPT compared to placebo, and the group treated with 1 kHz exhibited a reduction in temporal summation of pain compared to the other groups [21];
- Almeida et al. (2020) compared the analgesic effects of IFC therapy (2 and 4 kHz; 2 and 100 Hz) on subjects with chronic low back pain. One hundred and seventy-five subjects were randomly allocated to 1 of 5 groups (2 kHz, 100 Hz, sensory level; 2 kHz, 2 Hz, motor level; 4 kHz, 100 Hz, sensory level; 4 kHz, 2 Hz, motor level; placebo). IFC was applied with 4 electrodes on the lumbar area for 30 min. It was observed that 4 kHz with 100 Hz provided better analgesic effects in subjects with low back pain [22].

These studies demonstrated that the carrier frequency of 1 kHz with AMF of 100 Hz increased the PPT in healthy subjects [20], while carrier frequencies of 1 and 4 kHz with AMF of 100 Hz seem to promote higher analgesic effects, such as increased PPT and decreased analgesic consumption, pain intensity and temporal summation of pain in individuals with chronic low back pain [21,22]. In addition, the higher carrier frequencies (8 kHz or 10 kHz) present in some devices appeared to be less effective, albeit more comfortable [20].

### 6.2. Amplitude-Modulated Frequency (AMF)

The interference of two sinusoidal alternating currents can be constructive (waves in phase, producing a wave with a greater amplitude) and destructive (out-of-phase waves, when the rising segment of one coincides with the falling segment of another) [3]. The rate of resultant amplitude is equal to the difference in frequency between the two original waves and is called the “beat frequency” [3]. The amplitude-modulated frequency is a low-frequency current (1–250 Hz) generated by the interaction between two out-of-phase medium-frequency currents [23]. The literature on IFC therapy states that different parameters, such as the AMF, can cause different physiological effects (e.g., 130 Hz is more sedative; 0–100 Hz is more stimulating; 10–150 Hz increases blood flow; 50–100 Hz has sedative and spasmolytic effects) [24]. However, these claims seem to be based more on the personal and clinical experience of authors than on scientific evidence [24].

According to the literature, studies have been performed to investigate the analgesic and physiological effects of different AMFs in healthy subjects [23,24,25] and individuals with knee osteoarthritis [26] and chronic low back pain [22].

- Palmer et al. (1999) assessed the effects of different IFC and TENS frequencies on sensory, motor and pain thresholds in healthy subjects. Twenty-four women students received both IFC and TENS at different frequencies (IFC: 0, 5, 10, 15, 20, 30, 40 and 100 Hz; TENS: 5, 10, 15, 20, 30 and 40 Hz). Electrodes were positioned over the medium nerve and the current intensity was increased until sensory, motor and pain thresholds were reported. The peak current was recorded at each threshold for each frequency and averaged. According to the findings of this study, the IFC current did not produce a clear difference in current intensity in relation to the different types of AMF used. Moreover, there was no significant difference between pure 4 kHz stimulation (0 Hz AMF) and the other AMFs. On the other hand, TENS showed that lower frequencies require higher intensity to reach the threshold [25];
- Johnson and Tabasam (2003) investigated the analgesic effects of IFC with different AMFs on cold-induced pain in healthy subjects. Sixty individuals were randomly allocated to 1 of 6 IFC groups (20, 60, 100, 140, 180 and 220 Hz). A carrier frequency of 4 kHz was applied for 20 min, and the intensity was strong but comfortable with no visible muscle contraction. The time-to-pain threshold, pain intensity and pain unpleasantness were recorded pre-, during and post-treatment. No significant differences were found between groups for any outcome measures. Thus, the authors concluded that IFC therapy with different AMFs did not influence the analgesic effects on cold-induced pain in healthy subjects [24];
- Fuentes et al. (2010) examined the analgesic effects of IFC therapy with AMF on mechanically induced pain in healthy subjects. Forty-six healthy individuals received two applications of IFC (0 Hz and 100 Hz AMFs) in the lumbar area on two different days. The parameters used were 4 kHz for 30 min and a strong but comfortable sensory level intensity. PPTs in the lumbar area were evaluated pre-, during and post-application. There were no statistically significant intergroup differences. The addition of an IFC with an AMF does not seem to influence mechanical pain sensitivity in healthy subjects [23];
- Gundog et al. (2012) compared the effectiveness of different IFCs with AMFs on knee osteoarthritis. Sixty patients were randomized into 4 groups (40 Hz, 100 Hz, 180 Hz and placebo). A 4 kHz carrier frequency was used for 20 min, and the intensity was strong but comfortable. The patients received 15 treatments (5×/week), and the outcomes were pain intensity, disability, range of motion and paracetamol intake. The active groups were superior to the placebo, albeit with no statistical differences between them [26];
- Almeida et al. (2020) compared the analgesic effects of IFC therapy (2 and 4 kHz, 2 and 100 Hz) on subjects with chronic low back pain. One hundred and seventy-five subjects were randomly allocated to 1 of 5 groups (2 kHz, 100 Hz, sensory level; 2 kHz, 2 Hz, motor level; 4 kHz, 100 Hz, sensory level; 4 kHz, 2 Hz, motor level; placebo). IFC was applied on the lumbar area for 30 min using 4 electrodes. There were significant improvements in pain intensity in the active groups (2 kHz/2 Hz; 4 kHz/2 Hz; 4 kHz/100 Hz) compared to placebo and 2 kHz/100 Hz groups. In relation to the McGill Pain Questionnaire, the 4 kHz/2 Hz and 4 kHz/100 Hz groups showed better results compared to the placebo. For PPT, only 4 kHz/100 Hz was superior to placebo. In conclusion, 4 kHz/100 Hz provided better analgesic effects in subjects with low back pain [22].

In general, most of the studies performed in healthy subjects [23,24,25] and individuals with knee osteoarthritis [26] showed no differences in the analgesic effects of IFC therapy with different AMFs. However, Almeida et al. (2020) concluded that IFC therapy with 2 kHz/2 Hz significantly improved the pain intensity compared to 2 kHz/100 Hz in individuals with chronic low back pain [22].

### 6.3. Sweep Frequency (Delta F—∆F)

The sweep frequency means that the AMF can fluctuate between pre-determined upper and lower limits in an interchangeable manner [27]; in other words, ∆F is a variation of an AMF pre-set in the device. This means that with an AMF of 100 Hz and a ∆F of 50 Hz, the AMF variation will be between 100 and 150 Hz. This parameter is used to avoid sensory habituation [28]. However, Pivetta and Bertolini (2012) evaluated the duration of IFC habituation and how often it occurred in a crossover trial with 15 subjects. They received 10 min of IFC in the lumbar area with the following parameters: 4 kHz, 100 Hz (AMF), 1:1 (slope) and ∆F was adjusted according to the group (0 ∆F null = 0, ∆F low = 30% or ∆F high = 70%). There were no differences in habituation threshold or in how many times sensory habituation occurred between groups. Thus, it was concluded that the variation in AMF has no effect [28]. In addition, Dounavi et al. (2012) found no segmental or extrasegmental hypoalgesic effect using a pressure algometer to compare sweep AMF (80–110 Hz—within 6 s), constant AMF (110 Hz), placebo and control groups. A carrier frequency of 4 kHz (a strong and uncomfortable intensity) was applied for 30 min on the dominant forearm. The findings of this study were the same, regardless of the sweep AMF.

### 6.4. Sweep Mode (Slope) or Swing Pattern

**Figure 2:**
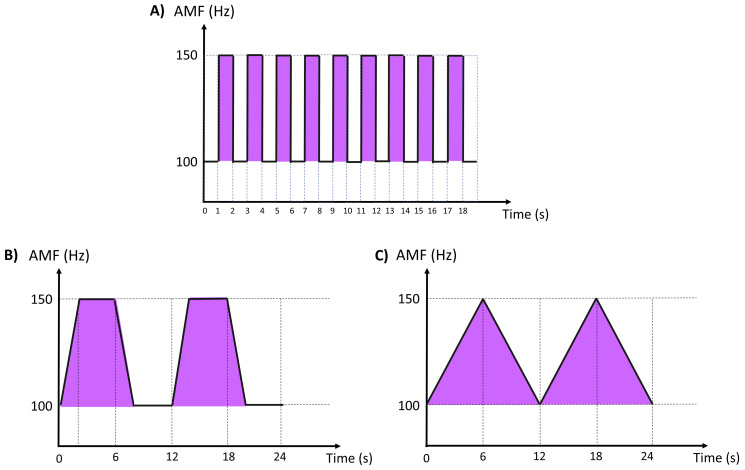
Sweep mode, slope or swing patterns: (**A**) 1:1—variation every 1 s; (**B**) 1:5:1—frequencies increase and decrease in 1 s and are maintained for 5 s; (**C**) 6:6—frequencies increase and decrease in 6 s; AMF: amplitude-modulated frequency; Hz: hertz; s: seconds.

This parameter is used to change the AMF between the lower and upper frequency limits on a fixed timeline [27]. The aim is to prevent the sensory habituation of the nervous system to repetitive electrical currents [27]. There are several types of swing pattern delivery ramps, such as 1:1 (variation every 1 s) (Figure 2A), 1:5:1 (frequencies increase and decrease in 1 s and remain stable for 5 s) (Figure 2B) and 6:6 (frequencies increase and decrease in 6 s) (Figure 2C) [29].

According to the literature, different swing patterns do not interfere in the analgesic effect or in sensory habituation in healthy subjects or individuals with low back pain, as described below:

- Johnson and Tabasam (2003) compared the analgesic effects of different IFC swing patterns on cold-induced pain in healthy subjects. Forty subjects were randomized into 1 of 4 treatment groups: burst, 1:1, 6:6 and 6∫6 (∫ = jumping, AMF remains at the lower frequency for 6 s before jumping to the upper frequency for 6 s). The IFC parameters were 4 kHz and an AMF of 100 Hz for 20 min with current intensity adjustment up to “strong but comfortable electrical paresthesia without visible muscle contraction”. The subjects completed 6 cycles of the cold-induced pain test: 2 pre-treatments, 2 during treatment and 2 post-treatments. The changes in pain threshold and pain intensity were evaluated. In conclusion, there were no intergroup differences in the hypoalgesic effects of different swing patterns [30];
- Adedoyin et al. (2005) examined the effects of different IFC swing patterns in subjects with low back pain. Thirty-nine subjects were allocated to three intervention groups based on three IFC patterns: 1:1, 6:6 or 6∫6 (∫ = jumping, AMF remains at the lower frequency for 6 s before jumping to the upper frequency for 6 s). The carrier frequency was fixed at 4 kHz and the AMF at 100 Hz for 20 min. Two electrodes, secured in place by Velcro straps and well-padded with lint, were positioned on the spinal nerve root corresponding to the painful area of the low back. The treatment was performed twice a day (2 times a week) for 3 weeks. No significant swing pattern differences were found for pain modulation in low back pain patients [27];
- Guerra and Bertoline (2012) evaluated the onset times of the first sensory habituation and the number of times it occurred during 10 min of IFC, varying the delta F (ΔF) delivery ramps. Eighteen healthy women were randomized into 3 groups: A (1:1; 1:5:1; 6:6), B (1:5:1; 6:6; 1:1) and C (6:6; 1:1; 1:5:1). IFC therapy was applied for 3 days according to the delta F specified. The IFC parameters were: 100 Hz of AMF, 50% of delta F and intensity above the sensory threshold. The first sensory habituation and how many times it occurred were recorded. There were no differences in sensory habituation threshold, although the 1:5:1 ramp had the lowest number of sensory habituations when compared to the 6:6 ramp [29].

### 6.5. Type of Application

IFC is applied transcutaneously with electrode pads (bipolar or quadripolar) [31].

#### 6.5.1. Bipolar Application

Two electrodes are used in this method, whereby amplitude modulation occurs within the stimulator [31] and the output is pre-modulated or exogenous IFC [1]. The signal leaving the equipment is modulated. With respect to bipolar application, the modulation depth in the tissue is the same in all directions—the modulation depth is always 100%.

#### 6.5.2. Tetrapolar (Quadripolar) Application

Four electrodes are used in this application, and interference occurs within the tissues [31]. It is also known as true or endogenous IFC [1]. The modulation depth depends on the direction of the currents and can vary from 0 to 100%. The region of maximum interference develops at 45 degrees diagonally between the two sets of electrodes, representing the region of therapeutic effect, which is static and situated deep in the tissues [3]. Since the tissues are not homogenous in relation to the electrical conductivity, the region of maximum stimulation tends to be more irregular [1].

#### 6.5.3. Tetrapolar (Quadripolar)—Automatic Vector Scan

The automatic vector scan makes it possible to increase the area of effective stimulation. The current intensity (amplitude) in the red circuit varies slowly between 50 and 100% of the maximum established value, and in the black circuit it is set automatically to 75% of the maximum current in the varying circuit. The direction in which the modulation depth is 100% depends on the ratio between the two currents. As a result, the area of maximum stimulation rotates back and forth in the region of intersection. Accurate positioning of the electrodes is important, since there are zones in which stimulation is not optimal. The patient must experience varying sensations of the current.

To date, we have found only two studies that have investigated the differences between bipolar and tetrapolar parameters:

- Ozcan et al. (2004) compared bipolar and tetrapolar applications and determined differences in the motor-to-sensory threshold ratio, maximum electrically induced torque and the comfort of each stimulation. Twelve healthy subjects received 4 different IFCs in a randomly allocated order: tetrapolar and crossed currents; bipolar and crossed currents; tetrapolar and parallel currents; bipolar and parallel currents. Four electrodes were attached to the right lower limb. A carrier frequency of 4 kHz and AMF of 50 Hz were used. According to the results, crossed currents did not show higher depth efficiency than their parallel counterparts, and bipolar application exhibited higher maximum electrically induced torque and less discomfort than its quadripolar counterpart. Thus, the authors concluded that tetrapolar is not superior to bipolar application in terms of the depth efficiency, torque production or comfort [1];
- Dounavi et al. (2012) performed a study to investigate the segmental and extrasegmental hypoalgesic effects of different IFC parameters on PPT in healthy subjects. One hundred and eighty healthy subjects were randomly allocated to 6 groups: control, placebo, bipolar constant AMF (110 Hz), bipolar sweep AMF (80–110 Hz), tetrapolar constant AMF (110 Hz), and tetrapolar sweep AMF (80–110 Hz). A frequency carrier of 4 kHz (strong and uncomfortable intensity) was used for 30 min on the dominant forearm. PPTs were measured on the first dorsal interosseous muscles on the dominant and nondominant hands (segmental measures) and the tibialis anterior muscle (extrasegmental measure) at baseline and at 10-min intervals. The results showed no significant differences in PPT between groups [31].

#### 6.5.4. Practical Applicability

The electrodes are positioned on the skin after being cleaned with soap and water to decrease linear electrical resistance, and arranged so that the two currents intersect in the treatment area [3]. Current intensity is increased until the patient reports that it is strong but comfortable [3].

#### 6.5.5. Contraindications

Patients with tumor, fever, acute inflammation, cardiac pacemaker, pregnant women and individuals with an aversion to electrical current therapy must be treated with caution [3].

## 7. Scientific Evidence of IFC

### 7.1. Systematic Reviews

We found 4 systematic reviews that investigated the efficacy of IFC for musculoskeletal pain [32,33] and knee osteoarthritis [9,34].

- Fuentes et al. (2010) analyzed the efficacy of IFC therapy in the management of musculoskeletal pain. Twenty studies were included. IFC therapy combined with other therapies seems to produce pain relief in acute and chronic musculoskeletal pain compared to no treatment or placebo. In patients with chronic low back pain, IFC therapy combined with other therapies was more effective than placebo at 3 months follow-up [32];
- Buenavente et al. (2014) performed a meta-analysis to evaluate the effectiveness of IFC on knee osteoarthritis. Four studies were included for meta-analysis. It was concluded that IFC therapy in conjunction with therapeutic exercise is effective in decreasing pain and paracetamol intake in subjects with knee osteoarthritis [9];
- Zeng et al. (2015) compared the efficacy of different electrical stimulation therapies (TENS, neuromuscular electrical stimulation (NMES), IFC, pulsed electrical stimulation (PES), and noninvasive interactive neurostimulation (NIN)) with a control group in the pain relief of subjects with knee osteoarthritis. Twenty-seven studies were included, and IFC was the only effective pain therapy when compared to controls. Thus, IFC therapy seems to be the best electrical stimulation option for pain relief in subjects with knee osteoarthritis [34];
- Hussein et al. (2021) analyzed the efficacy of IFC therapy in relieving musculoskeletal pain. Thirty-five trials were included, 19 of which were selected for meta-analysis. They concluded that IFC therapy alone reduced pain compared to placebo. Nevertheless, there were no significant differences between IFC and other interventions, such as laser, TENS or cryotherapy; IFC therapy plus standard treatment and placebo IFC therapy plus standard treatment; or IFC therapy plus standard treatment and standard treatment [33].

According to the systematic reviews presented, IFC therapy seems to be an effective analgesic electrical current, mainly for subjects with musculoskeletal pain or knee osteoarthritis.

### 7.2. Randomized Clinical Trials

Randomized clinical trials have been performed to evaluate the analgesic effects of IFC therapy in healthy subjects and those with pain disorders. The results in healthy subjects [31,35] are controversial. No positive IFC therapy results have been found for shoulder disorders [36,37]. Nevertheless, IFC therapy has shown analgesic effects for neck pain [38,39], low back pain [21,22,40,41,42], knee osteoarthritis [26,27,43,44,45] or postoperative knee pain [4,46]. Most of these studies have used the following parameters: carrier frequency of 4 kHz, AMF between 30–180 Hz, for 20–40 min with a strong but comfortable intensity. More details on these studies are presented in Table 1.

## 8. Conclusions

Clinical decisions about the use of IFC and its parameters should be based on scientific evidence. We hope that this review will be useful for physical therapists. IFC showed significant analgesic effects in patients with neck pain, low back pain, knee osteoarthritis and post-operative knee pain. Most IFC parameters seem not to influence the analgesic effect of this electrical current, except for carrier frequencies of 1 or 4 kHz with 100 Hz of AMF, which appear to promote greater analgesic effects than higher, more comfortable carrier frequencies. We encourage further studies to investigate the mechanism of action of IFC.

## Figures and Tables

**Table 1:** Randomized clinical trials of the effectiveness of IFC therapy.

Study	Groups (*n*)	F (kHz)	AMF (Hz)	∆ AMF	Sweep Mode	Time (Min)	Intensity	Sessions	Outcomes	Follow-Ups	Conclusion
IFC in Healthy Subjects											
Johnson 2002 [35]	IFC (10)	4	100	-	-	22	Strong but comfortable	1	Pain intensity	At baseline and post-treatment	**IFC was superior**
	Placebo IFC (10)	-	-	-	-		-				
	No treatment (10)	-	-	-	-	-	-				
Dounavi 2012 [31]	Bipolar constant (30)	4	110	-	-	30	Motor level	1	PPTs at the dorsal surface of the dominant and nondominant hand and tibialis anterior muscle	At baseline and post-treatment	No difference
	Bipolar sweep (30)	4	80	30	6:6	30	Motor level				
	Quadripolar constant (30)	4	110	-	-	30	Motor level				
	Quadripolar sweep (30)	4	80	30	6:6	30	Motor level				
	Placebo (30)	-	-	-	-	-	-				
	Control (30)	-	-	-	-	-	-				
IFC in Neck Pain											
Albornoz-Cabello 2019 [38]	IFC + EX (42)	4	60	90	-	25	Tolerance (adjustment, 3–5 min)	10 (5×/wk)	Pain intensity, disability, anxiety, depression, apprehension and ROM	At baseline and post-treatment	**IFC was superior**
	EX (42)	-	-	-	-	-	-				
Liu 2020 [39]	IFC (42)	5	200	Dynamic rhythm: 10 ± 2 s		30	Tolerance	5 consecutive days	Pain intensity, disability and hemodynamic indices	At baseline and post-treatment	**IFC + acupuncture was superior**
	Acupuncture (42)	-	-	-	-	-	-				
	IFC + Acupuncture (42)	-	-	-	-	-	-				
IFC in Shoulder Disorders											
Nazligul 2018 [37]	IFC (32)	4	100	-	-	20	Strong but comfortable (adjustment 5 min)	10 (5×/wk)	Pain intensity and disability	At baseline, post-treatment and after 1 month	No difference
	Placebo IFC (33)	-	-	-	-	-	-				
Gomes 2018 [36]	EX + MT + IFC	4	100	50	1:1	50	Strong but comfortable	16 (2×/wk)	Pain and disability (SPADI, NRS and pain-related self-statement)	At baseline and post-treatment	No difference
	EX + MT										
	EX + MT + Placebo US										
IFC in Low Back Pain											
Lara-Palomo 2012 [40]	Massage with IFC (31)	4	80	-	-	30	Motor level	20 (2×/wk)	Pain intensity, disability, fear of movement, resistance of abdominal muscles, lumbar flexion mobility	At baseline and post-treatment	**IFC was superior**
	Superficial manual massage (31)	-	-	-	-	-	-				
Correa 2016 [21]	IFC 1 kHz (50)	1	100	50	1:1	30	Strong, but comfortable (adjustment 5 min)	12 (3×/wk)	Pain intensity, disability, PPT, CPM, TS of pain, global perceived effect, discomfort and use of analgesics	At baseline, after 1st session and post-treatment	**IFC reduced use of analgesics and 1 kHz reduced TS of pain**
	IFC 4 kHz (50)	4	100	50	1:1	30					
	IFC Placebo (50)	-	-	-	-	-	-				
Franco 2017 [42]	IFC + Pilates (74)	4	100	50	1:1	30	Strong, but comfortable (adjustment 5 min)	18 (3×/wk)	Pain intensity, PPT, disability, global perceived effect and kinesiophobia	At baseline, post-treatment and after 6 months	No difference
	Placebo IFC + Pilates (74)	-	-	-	-	-	-				
Albornoz-Cabello 2017 [41]	IFC (44)	4	65	95	1:1	25	Sensorial level	10 (5×/wk)	Pain intensity	At baseline and post-treatment	**IFC was superior**
	Control (usual care) (20)	-	-	-	-	-	-				
Franco 2018 [47]	IFC + Pilates (74)	4	100	50	1:1	30	Strong, but comfortable (adjustment 5 min)	18 (3×/wk)	Pain intensity	At baseline and post-treatment	**IFC was superior**
	Placebo IFC + Pilates (74)	-	-	-	-	-	-				
Almeida 2020 [22]	IFC 1 (35)	2	100	0	-	30	Sensory level		Pain intensity and PPT	At baseline and post-treatment	**IFC 4 kHz/100 Hz provided analgesic effects.**
	IFC 2 (35)	2	2	0	-		Motor level				
	IFC 3 (35)	4	100	0	-		Sensory level				
	IFC 4 (35)	4	2	0	-		Motor level				
	Placebo (35)	-	-	-	-		-				
IFC in Post-operative of the knee											
Jarit 2003 [4]	IFC (46)	NR	5–10/80–150	-	-	14/14	Sensory level	3×/day for 7–9 wks	Pain intensity, edema, range of motion, use of pain medication	After 24 h, 48 h and 72 h, 1–9 weeks of the surgery	**IFC was superior**
	Placebo IFC (41)	-	-	-	-	-	-				
Kadi 2019 [46]	IFC (57)	NR	100	-	-	-	Strong but comfortable level	10 (2×/day)	Pain intensity, ROM, edema and use of paracetamol	At baseline, after 5 days and after 1 month	**IFC reduced paracetamol use on 5th day**
	Placebo IFC (56)	-	-	-	-	-	-				
IFC in Knee Osteoarthritis											
Adedoyin 2002 [48]	IFC (15)	NR	100/80	-	-	15/05	Sensory level	8 (2×/wk)	Pain intensity	At baseline and post-treatment	**IFC was superior**
	Placebo (15)	-	-	-	-	-	-				
Defrin 2005 [43]	IFC 1 (11)	4	30	30	-	20	Noxious unadjusted	12 (3×/wk)	Pain intensity, relief, and threshold; stiffness; ROM	At baseline and post-treatment	**IFC was superior to sham and control groups. Noxious stimulation was superior to innocuous**
	IFC 2 (11)	4	30	30	-		Noxious adjusted				
	IFC 3 (12)	4	30	30	-		Innocuous unadjusted				
	IFC 4 (11)	4	30	30	-		Innocuous adjusted				
	Sham (9)	-	-	-	-		-				
	Control (8)	-	-	-	-	-	-				
Gundog 2012 [26]	IFC 1 (15)	4	40	-	-	20	Strong but comfortable	15 (5×/wk)	Pain intensity, ROM, function, use of paracetamol	At baseline, post-treatment and after 1 month	**IFCs were superior to placebo**
	IFC 2 (15)	4	100	-	-						
	IFC 3 (15)	4	180	-	-						
	Placebo IFC (15)	-	-	-	-						
de Paula Gomes 2020 [44]	IFC + Ex (20)	4	75	25	1:1	40	Strong, but comfortable	24 (3×/wk)	Function, pain intensity, PPT, fatigue	At baseline and post-treatment	No difference
	PBM + Ex (20)	-	-	-	-	-	-				
	SWD + Ex (20)	-	-	-	-	-	-				
	Placebo + Ex (20)	-	-	-	-	-	-				
	Ex (20)	-	-	-	-	-	-				
Alqualo-Costa 2021 [45]	IFC + PBM (42)	4	50	50	1/1	30	Strong, but comfortable (adjustment 5 min)	12 (3×/wk)	Pain intensity, function, PPT, CPM and muscle strength	At baseline, post-treatment, after 3 and after 6 months	**IFC + PBM reduced pain intensity compared to placebo and isolated IFC at all time points**
	IFC + Placebo PBM (42)										
	Placebo IFC + PBM (42)	-	-	-	-	-	-				
	Placebo IFC + Placebo PBM (42)	-	-	-	-	-	-				

IFC: interferential current; MT: manual therapy; EX: exercises; wk: week; wks: weeks; *n*: number of subjects; F: frequency; AMF: amplitude-modulated frequency; Hz: hertz; kHz: kilohertz; min: minutes; PBM: photobiomodulation; SWD: shock-wave diathermy. Text marked in bold represents significant results between groups.

## References

[1] Ozcan J., Ward A.R., Robertson V.J. (2004). A comparison of true and premodulated interferential currents. Arch. Phys. Med. Rehabil..

[2] Ward A.R., Robertson V.J., Makowski R.J. (2002). Optimal frequencies for electric stimulation using medium-frequency alternating current. Arch. Phys. Med. Rehabil..

[3] Goats G.C. (1990). Interferential current therapy. Br. J. Sports Med..

[4] Jarit G.J., Mohr K.J., Waller R., Glousman R.E. (2003). The Effects of Home Interferential Therapy on Post-Operative Pain, Edema, and Range of Motion of the Knee. Clin. J. Sport Med..

[5] De-La-Cruz-Torres B., Martínez-Jiménez E., Navarro-Flores E., Palomo-López P., Abuín-Porras V., Díaz-Meco-Conde R., López-López D., Romero-Morales C. (2021). Heart Rate Variability Monitoring during Interferential Current Application in the Lower Back Area: A Cross-Sectional Study. Int. J. Environ. Res. Public Health.

[6] Jin H.-K., Hwang T.-Y., Cho S.-H. (2017). Effect of electrical stimulation on blood flow velocity and vessel size. Open Med..

[7] Ward A.R. (2009). Electrical Stimulation Using Kilohertz-Frequency Alternating Current. Phys. Ther..

[8] Johnson I.M., Tabasam G. (2003). An Investigation into the Analgesic Effects of Interferential Currents and Transcutaneous Electrical Nerve Stimulation on Experimentally Induced Ischemic Pain in Otherwise Pain-Free Volunteers. Phys. Ther..

[9] Buenavente M.L., Gonzalez-Suarez C., Lee-Ledesma M.A., Liao L. (2014). Evidence on the effectiveness of interferential current therapy in the treatment of knee osteoarthritis: A meta-analysis. OA Arthritis.

[10] Ariel E., Ratmansky M., Levkovitz Y., Goor-Aryeh I. (2019). Efficiency of Tissue Penetration by Currents Induced by 3 Electrotherapeutic Techniques: A Comparative Study Using a Novel Deep-Tissue Measuring Technique. Phys. Ther..

[11] Bellew J.W., Beiswanger Z., Freeman E., Gaerte C., Trafton J. (2011). Interferential and burst-modulated biphasic pulsed currents yield greater muscular force than Russian current. Physiother. Theory Pract..

[12] Ganne J.M. (1976). Interferential therapy. Aust. J. Physiother..

[13] Beatti A., Rayner A., Chipchase L., Souvlis T. (2011). Penetration and spread of interferential current in cutaneous, subcutaneous and muscle tissues. Physiotherapy.

[14] Noble G.J., Lowe A.S., Walsh D.M. (2000). Interferential Therapy Review. Part Mechanism of Analgesic Action and Clinical Usage. Phys. Ther. Rev..

[15] Melzack R., Wall P.D. (1965). Pain mechanisms: A new theory. Science.

[16] De Domenico G. (1982). Pain relief with interferential therapy. Aust. J. Physiother..

[17] Sánchez-Romero E.A., González-Zamorano Y., Arribas-Romano A., Martínez-Pozas O., Fernández Espinar E., Pedersini P., Villafañe J.H., Alonso Pérez J.L., Fernández-Carnero J. (2021). Efficacy of manual therapy on facilitatory nociception and endogenous pain modulation in older adults with knee osteoarthritis: A case series. Appl. Sci..

[18] Sánchez Romero E.A., Lim T., Villafañe J.H., Boutin G., Riquelme Aguado V., Pintado-Zugasti A.M., Alonso Pérez J.L., Fernández-Carnero J. (2021). The Influence of Verbal Suggestion on Post-Needling Soreness and Pain Processing after Dry Needling Treatment: An Experimental Study. Int. J. Environ. Res. Public Health.

[19] Johnson I.M. (1999). The Mystique of Interferential Currents When Used to Manage Pain. Physiotherapy.

[20] Venancio R.C., Pelegrini S., Gomes D.Q., Nakano E.Y., Liebano R.E. (2013). Effects of Carrier Frequency of Interferential Current on Pressure Pain Threshold and Sensory Comfort in Humans. Arch. Phys. Med. Rehabil..

[21] Corrêa J., Costa L., Oliveira N., Lima W., Sluka K., Liebano R. (2016). Effects of the carrier frequency of interferential current on pain modulation and central hypersensitivity in people with chronic nonspecific low back pain: A randomized placebo-controlled trial. Eur. J. Pain.

[22] Almeida N., Paladini L.H., Korelo R.I.G., Liebano R.E., De Macedo A.C.B. (2020). Immediate Effects of the Combination of Interferential Therapy Parameters on Chronic Low Back Pain: A Randomized Controlled Trial. Pain Pract..

[23] Fuentes C.J., Armijo-Olivo S., Magee D.J., Gross D. (2010). Does amplitude-modulated frequency have a role in the hypoalgesic response of interferential current on pressure pain sensitivity in healthy subjects? A randomised crossover study. Physiotherapy.

[24] Johnson I.M., Tabasam G. (2003). An investigation into the analgesic effects of different frequencies of the amplitude-modulated wave of interferential current therapy on cold-induced pain in normal subjects. Arch. Phys. Med. Rehabil..

[25] Palmer S.T., Martin D.J., Steedman W.M., Ravey J. (1999). Alteration of interferential current and transcutaneous electrical nerve stimulation frequency: Effects on nerve excitation. Arch. Phys. Med. Rehabil..

[26] Gundog M., Atamaz F., Kanyilmaz S., Kirazli Y., Celepoglu G. (2012). Interferential current therapy in patients with knee osteoarthritis: Comparison of the effectiveness of different amplitude-modulated frequencies. Am. J. Phys. Med. Rehabil..

[27] Adedoyin R.A., Ob Olaogun M., Onipede T.O. (2005). Effects of different swing patterns of interferential currents on patients with low back pain: A single control trial. Fiz. Rehabil..

[28] Pivetta K.M., Bertolini G.R.F. (2012). ΔF effects on the interferential current accommodation in healthy subjects. Ver. Bras. Med. Esporte.

[29] Guerra T.E.C., Bertolini G.R.F. (2012). Efeitos da variação da rampa de entrega do delta F sobre a acomodação da corrente interferencial em mulheres saudáveis. Ver. Dor..

[30] Johnson M.I., Tabasam G. (2003). A single-blind investigation into the hypoalgesic effects of different swing patterns of interferential currents on cold-induced pain in healthy volunteers. Arch. Phys. Med. Rehabil..

[31] Dounavi M.D., Chesterton L.S., Sim J. (2012). Effects of Interferential Therapy Parameter Combinations Upon Experimentally Induced Pain in Pain-Free Participants: A Randomized Controlled Trial. Phys. Ther..

[32] Fuentes J.P., Olivo S.A., Magee D.J., Gross D.P. (2010). Effectiveness of Interferential Current Therapy in the Management of Musculoskeletal Pain: A Systematic Review and Meta-Analysis. Phys. Ther..

[33] Hussein H.M., Alshammari R.S., Al-Barak S.S., Alshammari N.D., Alajlan S.N., Althomali O.W. (2021). A systematic review and meta-analysis investigating the pain-relieving effect of interferential current on musculoskeletal pain. Am. J. Phys. Med. Rehabil..

[34] Zeng C., Li H., Yang T., Deng Z.-H., Yang Y., Zhang Y., Lei G.-H. (2014). Electrical stimulation for pain relief in knee osteoarthritis: Systematic review and network meta-analysis. Osteoarthr. Cartil..

[35] Johnson M.I., Tabasam G. (2002). A single-blind placebo-controlled investigation into the analgesic effects of interferential currents on experimentally induced ischaemic pain in healthy subjects. Clin. Physiol. Funct. Imaging.

[36] Gomes C.A.F.d.P., Dibai-Filho A.V., Moreira W.A., Rivas S.Q., Silva E.d.S., Garrido A.C.B. (2018). Effect of adding interferential current in an exercise and manual therapy program for patients with unilateral shoulder impingement syndrome: A randomized clinical trial. J. Manip. Physiol. Ther..

[37] Nazligul T., Akpinar P., Aktas I., Unlu O.F., Cagliyan Hartevioglu H. (2018). The effect of interferential current therapy on patients with subacromial impingement syndrome: A randomized, double-blind, sham-controlled study. Eur. J. Phys. Rehabil. Med..

[38] Albornoz-Cabello M., Pérez-Mármol J.M., Quinta C.J.B., A Matarán-Peñarrocha G., Castro-Sánchez A.M., Olivares B.D.L.C. (2019). Effect of adding interferential current stimulation to exercise on outcomes in primary care patients with chronic neck pain: A randomized controlled trial. Clin. Rehabil..

[39] Liu Y., Qu L., Wang X., Xiong W., Li W., Abula H. (2020). Effects of warm acupuncture combined with stereo-dynamic interferential electrotherapy on clinical efficacy and hemodynamics of cervical spondylotic radiculopathy. Int. J. Clin. Exp. Med..

[40] Lara-Palomo I.C., Ferrandiz M.E.A., Matarán-Peñarrocha G.-A., Saavedra-Hernández M., Granero-Molina J., Fernández-Sola C., Castro-Sánchez A.M. (2012). Short-term effects of interferential current electro-massage in adults with chronic non-specific low back pain: A randomized controlled trial. Clin. Rehabil..

[41] Albornoz-Cabello M., Maya-Martín J., Domínguez-Maldonado G., Espejo-Antúnez L., Heredia-Rizo A.M. (2016). Effect of interferential current therapy on pain perception and disability level in subjects with chronic low back pain: A randomized controlled trial. Clin. Rehabil..

[42] Franco K.M., Franco Y.D.S., Oliveira N.B.d., Miyamoto G.C., Santos M.O., Liebano R.E. (2017). Is interferential current before pilates exercises more effective than placebo in patients with chronic nonspecific low back pain? A randomized controlled trial. Arch. Phys. Med. Rehabil..

[43] Defrin R., Ariel E., Peretz C. (2005). Segmental noxious versus innocuous electrical stimulation for chronic pain relief and the effect of fading sensation during treatment. Pain.

[44] de Paula Gomes C.A.F., Politti F., de Souza Bacelar Pereira C., da Silva A.C.B., Dibai-Filho A.V., de Oliveira A.R., Biasotto-Gonzalez D.A. (2020). Exercise program combined with electrophysical modalities in subjects with knee osteoarthritis: A randomised, placebo-controlled clinical trial. BMC Musculoskelet Disord..

[45] Alqualo-Costa R., Thomé G.R., Perracini M.R., Liebano R.E. (2018). Low-level laser therapy and interferential current in patients with knee osteoarthritis: A randomized controlled trial protocol. Pain Manag..

[46] Kadı M.R., Hepgüler S., Atamaz F.C., DeDe E., Aydoğdu S., Aktuglu K., Ozkayın N., Ozturk C. (2019). Is interferential current effective in the management of pain, range of motion, and edema following total knee arthroplasty surgery? A randomized double-blind controlled trial. Clin. Rehabil..

[47] Franco Y.R., Franco K.F., Silva L.A., Silva M.O., Rodrigues M.N., Liebano R.E. (2018). Does the use of interferential current prior to pilates exercises accelerate improvement of chronic nonspecific low back pain?. Pain Manag..

[48] Adedoyin R.A., Olaogun M.O., Fagbeja O.O. (2002). Effect of Interferential Current Stimulation in Management of Osteo-arthritic Knee Pain. Physiotherapy.

